# Brain, Cognition, and Psychoanalysis: A Scoping Review

**DOI:** 10.3390/brainsci15060562

**Published:** 2025-05-24

**Authors:** Anna Rita Giovagnoli, Panayiotis Patrikelis, Annalisa Parente, Alessandra Parisi, Rute Flavia Meneses

**Affiliations:** 1 Unit of Neurology and Neuropathology, Foundation IRCCS “Carlo Besta” Neurological Institute, Via Celoria 11, 20133 Milano, Italy; annalisa.parente@istituto-besta.it (A.P.); alessandra.parisi@istituto-besta.it (A.P.); 2 Laboratory of Neuropsychology and Behavioral Neuroscience, Department of Psychology, Aristotle University of Thessaloniki, University Campus, 54124 Thessaloniki, Greece; ppatrikelis@gmail.com; 3 Faculty of Humanities and Social Sciences, RISE-Health, Fernando Pessoa University, Praça de 9 de Abril 349, 4249-004 Porto, Portugal; rmeneses@ufp.edu.pt

**Keywords:** neurology, memory, language, executive functions, brain connectivity, psychoanalytic psychotherapy

## Abstract

Background: Cognitive functions and brain connectivity could be influenced by psychoanalytic psychotherapy (PP), thus representing neurobiological parameters for therapy-induced changes. This study searched empirical studies on cognition and the brain to evaluate which functions have been assessed, with which instruments, and what changes have been documented in brain connectivity after PP. Methods: We used the guidelines and checklist of the Preferred Reporting Items of Systematic Reviews and Meta-analyses Extension for Scoping Reviews. The literature search was performed on the Medline–PubMed, American Psychological Association-PsycINFO, Elton Bryson Stephens Company, and Cochrane databases, and Google Scholar, including articles on patients with non-psychotic disturbances published from 1980 to September 2024. Results: Fifty-nine articles were collected. Five articles reported on cognitive outcomes. Abstraction and mentalization remained stable after individual PP in patients with adjustment disorders or anorexia nervosa. Executive functions, emotional intelligence, spatial short-term memory, attention, and balance between relatedness and self-definition improved after group PP applied alone or combined with individual PP. Twelve studies using functional magnetic resonance imaging, positron emission tomography, single-photon emission computerized tomography, or electroencephalography showed functional brain changes after different types of PP. Conclusions: An empirical approach has rarely been used to evaluate the impact of PP on the brain and cognition. The results of selected studies on neurotic and depressive disorders suggest that PP can stimulate cognitive function and brain connectivity. Further literature reviews are needed to clarify these issues and provide an avenue for research studies targeting PP in different conditions. Communication between neurology and psychoanalysis is indispensable.

## 1. Introduction

Since the 1990s, various articles have addressed the link between neuroscience and psychoanalytic psychotherapy (PP), reporting studies on consciousness, attachment, affect regulation, self-regulation, and patient–therapist relations (see for instance, [1,2,3]). This link has guided neuroscientific research towards a better understanding of psychodynamic concepts, such as the defense mechanisms, dream functions, or projection of mental states, and there has been cooperation to enhance knowledge on memory, awareness, empathy, trauma, attachment, the Self and the Ego, and the part of the personality that thinks and acts through the body on a reality plane, based on genetic and epigenetic factors such as caregiving, attachment style, coping strategies, and resilience [4].

Early studies also suggested that psychotherapy can modify brain circuits [5] and that the neurobiological effects of PP are important for its theoretical models [6]. Since the circuits underlying implicit and explicit cognitive functions extend over numerous neural systems, they may be especially susceptible to PP, so cognitive modifications after PP may express changes in brain activity. Implicit cognitive functions are automatic, fast, and unconscious, while explicit functions are slow and linked to awareness, reflection, and control over thoughts and actions, with impact on emotion regulation [7]. Schore’s [8,9,10] model addressed the role played by the right hemisphere in self-regulation, the encoding of mental patterns, the memories below the level of awareness, and the interaction between implicit and explicit cognitive functions since early life. During the second and third year of life, the development of language and abstraction provides a basis for explicit functions such as reasoning, focused and sustained attention on goal-ended actions, learning, episodic and semantic memory, risk monitoring, decision making, and theory of mind (ToM) [11]. Hence, the early biological development of the right hemisphere underlies the development of implicit functions, while the subsequent development of the left hemisphere supports explicit functions, personal identity, and interpersonal relations. The encounter between implicit cognitive schema stored deep in the brain structure (e.g., basal ganglia) and conscious reflection supported by cortical networks (e.g., the left frontotemporal cortex) is important for self-regulation and relations throughout the lifespan. Intuitively, communication between the therapist and the patient or members of a therapeutic group involves both implicit and explicit cognitive functions. The interplay of these and environmental dimensions can favor reflection on past perspectives and the Self and help bring unconscious emotions and defensive mechanisms to light [10], thus complementing therapy. Based on Siegel’s [12] interpersonal neurobiology theory, primary interactions with the mothering person influence the structure of the limbic and cortical regions, generating implicit memories that will influence the value and expectations attributed to relationships with others during adulthood. These relational patterns are deeply rooted and undetectable, so they are reproduced even within the therapy group because they are implicitly accepted as the truth. This primordial form of memory tends to evolve; around the second year of life, a process of integration between implicit and explicit memory begins. In the developing limbic system, the amygdala (central implicit memory) links with the hippocampus (mapper of explicit memory) to generate a whole experience with spatial–temporal markers. The limbic areas subsequently begin to form connections with the medial prefrontal cortex, first in the right hemisphere (sense of the Self in one’s story) and then in the left hemisphere (verbal account of one’s story) underlying autobiographical memory. Hence, the human mind develops through a mutual influence framework between interpersonal relations and brain anatomy and physiology.

Cognitive changes have been considered biomarkers of brain plasticity induced by stimulation in psychotherapy and cognitive rehabilitation (see for [13,14,15]). Kandel [16] was the first to highlight that learning processes can permanently modify and reinforce synaptic connections. Based on research on gene–environment interaction, Kandel [17,18] theorized a biological approach in which psychotherapy is conceived of as a learning process that can determine changes in genes and modify the strength of synaptic connections. Margulies et al. [19], based on functional magnetic resonance (fMRI), showed negative relationships between anterior cingulated (ACC) affective networks and dorsocaudal ACC attention networks. Subsequent studies demonstrated that successful talk-based interventions may lead to significant brain changes [20], and that free association in healthy individuals can induce neural responses detected by fMRI [21,22].

In neurological patients undergoing rehabilitation [23,24,25,26,27], the assessment of cognitive functions has provided information on treatment-induced changes. In patients undergoing PP, such an assessment could also highlight certain therapeutic effects.

Overall, within this framework, the assessment of cognitive functions in patients undertaking PP appears reasonable and worthy of an empirical approach. In this regard, in the 1900s, Doidge [28] underlined the usefulness of an empirical approach for evidencing the effects of PP. This empirical perspective has been particularly addressed in the field of PP, as, based on different approaches, it has been shown to stimulate long-lasting affective and cognitive improvements more effectively than non-psychoanalytic psychotherapy [28]. The empirical approach is in line with the World Health Organization’s [29] guidelines, which defined the outcome as a “change in the health of an individual, group of people, or population that is attributable to an intervention or series of interventions” and gave indications to provide transparent measures of therapies’ results. Evaluating PP outcomes would provide evidence-based information useful for clarifying its influence on brain function, for sharing knowledge among professionals, and for comparing psychotherapies with different settings and durations or the benefits of PP and other therapies. It is important to note that the outcomes of PP are complex and prolonged over time and include a variety of changes such as the alleviation of symptoms, improved mentalization, awareness of defensive mechanisms, self-identification, increased empathy, and the deepening of the therapeutic relationship. Such changes may be appreciated by patients and therapists, but may be difficult to understand outside the clinical context. Therefore, an empirical approach to PP may be important to share evidence-based effects.

In short, brain activity can be influenced by PP and cognitive functions can represent parameters for therapy-induced changes. An empirical design for the study of PP outcomes has previously been advocated, and cognitive assessment can collaborate in such a design. On this basis, it appears to be not only of speculative interest, but also necessary as a prerequisite for clinical studies, to evaluate the current knowledge based on the link between the brain, cognitive functions, and PP. The aim of this study was to examine articles on cognitive and brain changes after individual or group PP. The specific aims were to assess which cognitive functions have been assessed, with which instruments, and what changes have been documented in cognitive functions and brain connectivity.

## 2. Materials and Methods

### 2.1. Design

Given the mixed conceptual framework of this review and the irregular empirical approach to PP over time, a degree of scope clarity was required that is incompatible with the narrow and rigorous methodology of systematic approaches. A scoping review was the best-suited literature review method to evaluate current the knowledge about cognitive functions and brain connectivity in patients undergoing PP. There is no absolute minimum number of articles needed for a scoping review. For instance, the National Library of Medicine (National Institute of Health) does not specifically address such an issue, while the Becker Medical Library (Washington University School of Medicine) specifies that the number of articles retrieved depends on a variety of factors such as how narrow/wide the research question is and the novelty of the topic.

This review was developed using the methodological framework proposed by Peters [30,31], the guidelines suggested by Arksey and O’Malley (2005) [32], and the checklist of the Preferred Reporting Items of Systematic Reviews and Meta-analyses Extension for Scoping Review [33]. According to the objectives, this review was divided into two parts: cognitive functions in individual and group PP and brain connectivity.

### 2.2. Research Questions

The central questions of this study were as follows:

- Which cognitive functions are evaluated in patients undergoing PP?
- Which measurements are used to evaluate the cognitive outcomes of PP?
- Which functional brain changes follow PP?

### 2.3. Eligibility Criteria

The titles, keywords, and abstracts of the articles were analyzed to see if they matched the selection criteria. The inclusion criteria were as follows: articles published in English, original research with at least one measurement referring to PP, participants with psychopathological symptoms in the category of neurosis, depression, and anxiety (S2 and S3 sections of the Symptom patterns of the Psychodynamic Diagnostic Manual (PDM-2) [34], participants older than 18 years of age, peer-reviewed articles/journals, and the presence of PP in the title, keywords, or abstract. Given that single cases are usually discussed in PP supervisions and inter-visions, articles dealing with single cases or small groups were selected along with observational studies and controlled randomized studies. Articles dealing with psychosis, stress-related disturbances, disturbances related to physical diseases, specific symptoms, disturbances related to substances abuse, and medical conditions, particular psychological experiences (sections S1 and from S4 to SApp of the PDM-2 symptom patterns), as well as book chapters, congress abstracts, reviews or meta-analyses and studies with participants with psychosis, borderline disorders, additional medical disorders, or undergoing neurosurgery were excluded. Conversely, the review’s section on brain functions considered all symptom patterns of the PDM-2.

### 2.4. Search Strategy

The electronic literature search was conducted on the Medlin–PubMed, American psychological Association (APA)-PsycINFO, Elton Bryson Stephens Company (EBSCO), and Cochrane Collaboration databases, and Google Scholar, using the advanced search and punctuation options for each database/engine and for the following terms:

- Individual PP: (Cognitive functions OR Cognition OR Cognitive improvements OR Cognitive reserve OR Memory OR Language OR Attention OR Executive functions OR Social Cognition OR Theory of mind OR Emotion recognition OR Neuropsychological tests) AND individual PP/psychodynamic psychotherapy.
- Group PP: (Cognitive functions OR Cognition OR Cognitive improvements OR Cognitive reserve OR Memory OR Language OR Attention OR Executive functions OR Social Cognition OR Theory of mind OR Emotion recognition OR Neuropsychological tests) AND group PP/psychodynamic psychotherapy.

Identified records were excluded before screening based on the criteria described above and if methodological details were not available.

The search for articles regarding PP and brain connectivity was implemented in two steps, with the latter using the names of measurement instruments.

The first step used the following keywords:

- (Brain OR Brain Connectivity OR Brain Plasticity) AND PP/psychodynamic psychotherapy.

The second step used the following keywords:

- PP/psychodynamic psychotherapy AND Positron emission tomography (PET) OR Single photon positron emission computerized tomography (SPECT) OR fMRI OR Electroencephalography (EEG).

The data search took place from May 2022 to September 2024; it was not simultaneous in all databases and, given the long duration and complexity of the topic, was repeated over time to ensure the verification of new articles. A two-screen single assessment was used to ensure that the inclusion and exclusion criteria were met: (1) preliminary screening, consisting of an analysis of titles, keywords, and abstracts; (2) eligibility testing, consisting of a full-text screening. The data from the selected articles were reported in a data extraction model that included (1) publication aspects: first author, year of publication, and state in which the study was conducted; (2) study characteristics: type of study, study purpose, and neuropsychological instruments; (3) type of participants: sample size, age range, age at disease onset, duration of disease, and number of psychopharmacological drugs; and (4) relevant outcomes. Quality assessment of the selected studies was not conducted as it was beyond the scope of this review and is not a requirement of a scoping review.

## 3. Results

### 3.1. Individual Psychoanalytic Psychotherapy

**Figure 1:**
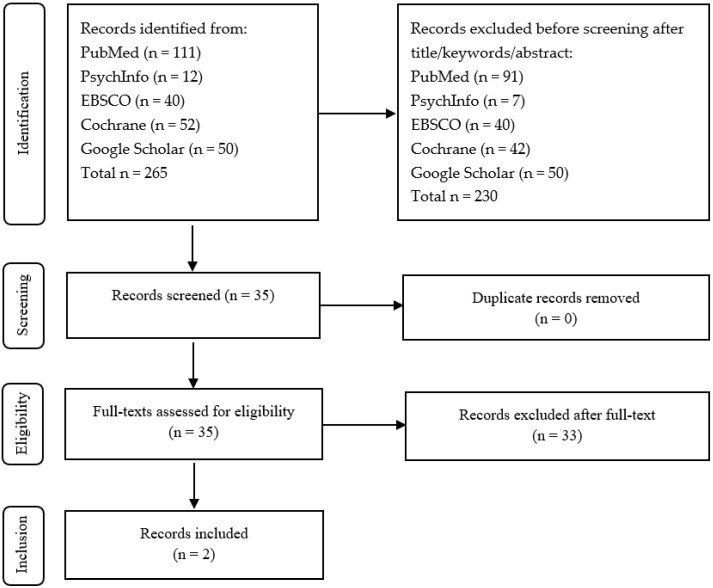
Flow chart of the review on psychoanalytic individual psychotherapy.

Two hundred and sixty-five articles published before July 2024 were identified. The first review excluded 230 articles (reviews, editorials, citations, research protocols, and articles reporting experimental studies without cognitive outcomes, or dealing with childhood and adolescence). Three articles dealing with longitudinal naturalistic studies were identified [35,36,37]. Out of these, two studies only involving individual PP were selected, [35,36] and one [37] was reassigned to the category of PP using both the individual and group settings (Figure 1, Table 1).

Kramer et al. [35] reported on the outcome of cognitive biases in patients with adjustment disorders treated via individual PP. These authors applied short-term PP lasting up to a year, with a mean of 34 sessions. Cognitive outcomes focused on cognitive errors (fortune-telling, overgeneralization, selective abstraction, and personalization) committed during therapeutic sessions. Assessment was undertaken by means of the Cognitive Errors Rating Scale [38], compiled by external raters based on transcripts of interviews, at three points of the therapy: early (session 4–7), mid-treatment (session 12–17), and close to the end (after the 20th session). The results showed a decrease in selective abstraction and an increase in personalization and suggested that these changes may have a protective function in psychotherapy. Zeek et al. [36] evaluated the capacity to mentalize (reflective function) in patients with anorexia nervosa by means of the In-Session Reflective Functioning Scale [39]. In this study, there was no increase in mentalization capacity during individual PP, but mentalization was associated with symptom improvements in the last phase of the therapy.

### 3.2. Group Psychoanalytic Psychotherapy

**Figure 2:**
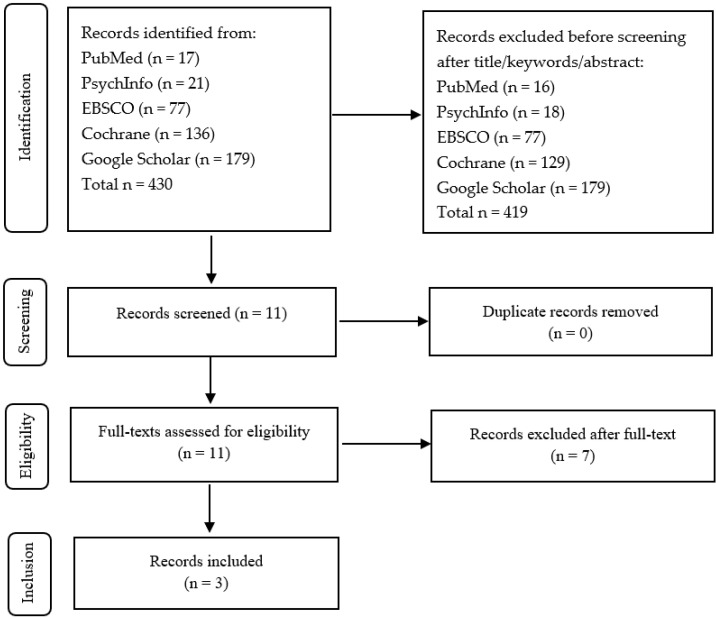
Flow chart of the review on psychoanalytic group psychotherapy.

Four hundred and thirty articles published before Decembre 2023 were identified. The first review excluded eleven articles because they did not deal with the target topic, while a further two articles were excluded due to the type of pathology (traumatic brain injury patients, multiple sclerosis, and schizophrenia) (Figure 2). Three articles [37,40,41] were selected and underwent full text analysis. Of these, Forghani et al.’s article [40] only dealt with group PP (Table 2), while the others [37,41] included both individual PP and group PP (Table 3).

Forghani et al. [40] analyzed the effect of group psychotherapy on emotional intelligence (EI) and executive functions in addicts. The tools used were the Emotional Quotient Inventory (EQ-i) [42] to evaluate EI, and the Stroop Test [43] to evaluate executive functions and selective attention (the ability to inhibit cognitive interference as expressed in response competition). The assessment was at the beginning of the treatment and then after 12 sessions of therapy. At the end of the treatment, the results showed significant improvement in both executive functions and EI.

Klasik et al. [37] evaluated cognitive outcomes in patients treated via individual plus group PP. This study analyzed short-term memory and attention deficits in patients treated for depressive disorders. The study compared three groups: the first treated only with psychodynamic psychotherapy, the second received both psychotherapy and pharmacological treatment, and the third was treated only pharmacologically with sertraline. In addition to individual psychotherapy, patients participated in group psychodynamic meetings. The main tools for cognitive functions were the Corsi tapping test to measure the short-term visual–spatial memory span and the Signal Detection test to measure the visual discrimination ability and selective attention [44]. The assessment was at the beginning of the treatment and after 8 weeks of therapy. At the end of the treatment, the greatest improvement in short-term memory and attention processes was observed among patients who received combined individual and group psychotherapy, compared to those treated only with psychotherapy or pharmacotherapy. Werbart [41] evaluated the cognitive outcomes in patients treated with individual or group PP. This study included, in particular, 42 patients undergoing group PP for an average of 16 months and 47 patients undergoing individual PP for an average of 25 months. Patients were assessed by means of Blatt’s object relations inventory [45] for self-representation and the Differentiation Relatedness Scale [46], showing an improvement in balance and self-definition and a better integration of self-representation in patients treated with group PP. Table 4 summarizes the neuropsychological measurements in studies on individual and group psychoanalytic psychotherapy.

### 3.3. Brain Connectivity and Psychoanalytic Psychotherapy

Table 5 summarizes the results of 12 studies on the association between PP and brain function as assessed by means of fMRI, PET, SPECT, and EEG (three studies each).

Beutel et al. [47] used an emotional linguistic go/no-go fMRI design to determine changes in the brain activation patterns of panic disorder after short-term psychodynamic inpatient treatment. The results revealed changes in frontal limbic circuitry, not dissimilar to those previously observed after cognitive behavioral treatments. De Greck et al. [48] in an fMRI study employing a face emotional recognition paradigm, tested whether para-hippocampal gyrus activity normalized after (inpatient) multimodal PP in somatoform disorder (SD) patients. All patients participated in a standardized patient multi-modal PP, including individual PP, group PP, medical therapy, and other methods (music therapy, communicative movement therapy, art therapy, social therapy, and relaxation methods). PP aimed to improve the verbalization of emotional and interpersonal problems, to improve affect perception, and to enhance the understanding of the intra-psychic and interpersonal conflicts underlying the patient’s symptoms. Since the involvement of the para-hippocampal gyrus in emotional memory, it was assumed that its decreased activation in SD (to defend against overwhelming emotions) may reflect a process of repression over emotional memories. Increased hemodynamic responses in the para-hippocampal gyrus bilaterally, as well as in other regions following multimodal PP, provide a neurophysiological key to understanding SD in terms of decreased PG activation. Wiswede et al. [49] evaluated brain functional responses to individualized stimuli in patients with major depression eight months after PP and compared them to control subjects. At baseline, patients showed enhanced activation compared to controls in several limbic and subcortical regions, including amygdala and basal ganglia. After PP, the differences in brain activity between patients and controls were no longer evident, while patients showed significant improvements in depression.

Among the studies using PET, Karlsson et al. [50,51] compared PP and selective serotonin reuptake inhibitor drug therapy in major depression. These studies showed that increased 5-HT 1A receptor density after psychotherapy was strongly associated with increased social and occupational functioning. In another patient group with major depression, Hirvonen et al. [52] compared the clinical and neurobiological effects of short-term PP and fluoxetine by testing the ventral striatal D2/3 receptor binding with raclopride in a PET scan. This study showed no change in D2/3 receptor binding in this area, neither after fluoxetine nor PP treatment.

Studies that used SPECT include single case studies. Tolmunen [53] reported on a case characterized by simultaneous symptoms of major depression and hypomania. The patient had an elevated serotonin transporter availability (SERT) in the mid-brain and elevated dopamine transporter availability (DAT) in the striatum, which normalized at the one-year follow-up after eight months of PP. Saarinen [54] explored the outcome of PP in a patient with major depression by analyzing the serotonin transporter binding at baseline and at the one-year follow-up. Lehto [55] studied changes in neurotransmitter transporter levels in depressed patients. While all patients showed a significant decrease in symptoms after one year of PP, as measured with depression scales, a significant increase in SERT levels was only seen in patients with atypical depression.

**Table 5:** Studies on brain functioning after psychoanalytic psychotherapy.

Reference Country	Aim	Participants Diagnosis	Demographics *	Cognitive and Psycho-Behavioural Measurements
Magnetic resonance				
Beutel et al. 2010 Germany [48]	To assess functional brain changes during short-term PP using MRI 1.5T	9 patients panic disorder 18 healthy subjects	Age: patients: 32 years healthy subjects: 29 years Schooling not reported	Emotional linguistic go/no-go task
De Greck et al., 2013 Germany [49]	To assess functional brain changes after PP using fMRI.	15 patients somatoform disorder	Age: 42.6 years Schooling not reported	Toronto Alexithymia Scale-20 Symptom check list 90 Beck Depression Inventory Tübinger Affekt Batterie
Wiswede et al. 2014 Germany [50]	To assess functional brain changes during long-term PP using MRI 3T	18 patients Major Depression 17 healthy subjects	Age: patients: 39.8 years, healthy subjects: 38 years Schooling not reported	Operationalized Psychodynamic Diagnostics, traffic and relaxation sentences task
Positron emission tomography				
Karlsson et al., 2010 Finland [51]	To test and compare the effects of fluoxetine (selective serotonin reuptake inhibitor) and short-term PP on 5-HT1A receptor density, binding potential, in patients with major depressive disorder	23 patients Major depression: 8 treated with PP 15 treated with Fluoxetine 4 healthy subjects	Age: PP group: 41 years Fluoxetine group: 39 years Schooling: PP group: 1.75 years Fluoxetine group: 1.80 years	17-item Hamilton Depression Rating Scale Beck Depression Inventory
Karlsson et al., 2013 Finland [52]	To compare the relationship of increased serotonin receptor 1A binding with social functioning in major depressive disorder after PP and serotonin treatment	23 patients Major depression: 8 patients treated with PP 15 treated with Fluoxetine 4 healthy subjects	Age: PP group: 41 Fluoxetine group: 39 Schooling: PP group: 1.75 years, Fluoxetine group: 1.80 years	Hamilton Depression Rating Scale Beck Depression Inventory Social and Occupational Functioning Assessment Scale Social Adjustment Scale-Self-Report Brief Symptom Inventory
Hirvonen et al., 2011 Finland [53]	To test whether fluoxetine and short-term PP increase D_2/3_ receptor binding assessed with raclopride PET in patients with major depressive disorders	22 patients Major depression: 8 patients treated with PP 14 patients treated with Fuoxetine	Age: PP group: 41 years, Fluoxetine group: 39 years Schooling: PP group: 1.75 group, Fluoxetine group: 1.79 years	17-item Hamilton Depression Rating Scale Beck Depression Inventory
Single photon emission computerized tomography				
Tolmunen et al. 2004 Finland [54]	To evaluate serotonin and dopamine transporter densities in mania or hypomania after PP	1 female patient with hypomania and dysthymia 6 depressed patients 10 healthy subjects	Age: patient with hypomaniaand and dysthyimia: 25 years, depressed patients: 27.2 years, healthy subjects: 26.3 years Schooling not reported	Hamilton Depression Rating Scale
Saarinen et al., 2005 Finland [55]	To explored the outcome of PP of a female patient with major depression using clinical evaluation and serotonin transporter binding assessed with [^123^I]nor-β-CIT	1 female patient Depression 10 healthy subjects	Age: patient: 20 years, healthy subjects: 26.3 years Schooling not reported	Hamilton Depression Rating Scale Montgomery Asberg Depression Rating Scale
Lehto et al., 2008 Finland [56]	To investigate the effect of PP on serotonin and dopamine transporter functions in depressed subjects	19 patients Depression: 8 with atypical depression 11 with typical depression)	Age: atypical group: 28.2 years, typical group: 27.1 years Schooling not reported	Hamilton Depression Rating Scale
Electroencephalography				
Utterrainer et al. 2014 [57]	To explore the clinical effects of short-term PP combined with bio-feedback and related EEG changes	Multiple drug misuse	19-year-old male patient One university year	Brief Psychiatric Rating Scale Montgomery-Asberg Depression Rating Scale
Buchheim et al. 2018 [58] Austria	To seek neurophysiological changes associated with improvements during PP PP was administered by 16 highly experienced psychoanalysts	17 patients Depression: 8 with two types of depression 10 with comorbid anxiety 13 healthy subjects	Age and schooling: not reported	Adult Attachment Projective Picture System Adult Attachment Interview Beck Depression Inventory
Buchheim et al. 2023 USA [59]	To evaluate the neurophysiological changes with contemporary electroencephalogram recording	1 patient Major depression, borderline personality disorder	Age: 27 years Schooling: not reported	Structure interview conducted with standardized format

* Mean age and schooling. PP, psychoanalytic psychotherapy. EEG, electroencephalograpfhy. PET, positron emission tomography. SPECT, single photon emission computerized tomography.

A few studies examined neural changes during PP using EEG [56,57,58]. Unterrainer et al. [56] explored the real-time relationship between psychodynamic clinical processes and their manifestation in neural processes by integrating neurofeedback in PP sessions. A 19-year-old male, affected by polydrug misuse for 18 months, underwent short-term psychodynamic psychotherapy combined with neurofeedback and EEG recording. Follow-up showed a contemporary clinical improvement in psychopathological symptoms, in particular in feelings of anhedonia and alienation, and a reduction in the theta potential’s amplitude. Buchheim et al. [57] demonstrated that, at the beginning of the PP, patients showed significantly higher late positive potentials (LPPs) in the frontocentral brain areas and sustained gamma-band activity compared to the controls. After 15 months of therapy, in the patient group, the LPP amplitudes and gamma-band responses decreased and equalized those registered in healthy controls. Here, the LPP and gamma-band activity were considered potential endophenotypes of the processing of emotions over the PP course. In a 27-year-old male patient with major depression and borderline personality disorder, with recurrent suicidal and self-injurious behavior, Buchheim et al. [58] investigated the neural correlations of PP interventions by analyzing the EEG power spectrum. Three different psychoanalytic interventions, based on clarification, confrontation, or interpretation, and a neutral control condition, emerging from a structured psychoanalytic interview during EEG recording, were compared. Two independent experts selected the experimental conditions within the interview, while fast Fourier transformation (FFT) was applied for analyzing the EEG spectra in central, frontal, and parietal areas corresponding to each intervention segment. In comparison with the control condition, the interpretation and confrontation interventions showed significantly lower alpha power in the central brain areas, whereas no significant effects were observed in the frontal and parietal areas for the alpha or beta bands. The authors suggested that PP interventions, particularly when involving the interpretation of unconscious intrapsychic conflicts, may change psychic processes, consistent with the effects on alpha power.

## 4. Discussion

Brain activity can be influenced by PP, and cognitive functions can represent parameters for therapy-induced changes. An empirical design for the study of PP outcomes has previously been advocated. This scoping review examined the current knowledge on cognitive functions and brain connectivity after PP to determine which functions have been assessed, with which instruments, and what changes have been documented in brain connectivity.

### 4.1. Cognitive Functions Evaluated in Patients Undergoing Psychoanalytic Psychotherapy

The results evidence that, since the 1980s, 59 studies have investigated the link between cognition, the brain, and psychoanalysis, or used an empirical approach to PP. Out of these, five investigated cognitive outcomes in patients affected by adjustment disorders, depression, anxiety, or anorexia. In selected studies, from three months to three years after PP, patients improved in cognitive bias, abstraction and personalization [35], mentalization [36], interference control and emotional intelligence [40], short-term visual–spatial memory and selective attention [37], or relatedness and self-representation [41]. Qualitative comparisons of the results of studies that used individual PP, group PP, or both show that abstraction and mentalization remained stable after individual PP in patients with adjustment disorders or anorexia nervosa. In contrast, executive functions, emotional intelligence, short-term spatial memory, attention, relatedness, and self-definition improved when group PP was applied alone or combined with individual PP in patients with depression, anxiety, or personality disorders. The non-comparability of diagnoses, assessed functions, and therapy contexts and times prevents a quantitative account establishing the prevalence of group PP benefits, although it is to be expected that group PP can improve cognitive functions due to the multiplicity and variety of stimuli arising from the group. In fact, group PP has been found to be more effective than individual psychotherapy in several clinical conditions [59]. Its added value compared to individual therapy is a real-life situation [60]. It offers the opportunity to use social interactions to explore and share inner worlds, as well as to provide mutual support in a safe environment [61]. These interactions can stimulate understanding of other participants’ feelings, emotions, and achievements, enhancing one’s awareness of one’s own problems and personal development. In addition, interactions with different personalities can evoke diverse reactions, new relational patterns, and an increased awareness of reality. It is known that brain plasticity is enhanced by an environment enriched by physical and social activities; in this condition, an increase in synaptic contacts, branching, dendritic spines, and neuron size has been observed compared to in impoverished environments [62].

Although the cognitive facets of PP were addressed early in Freud’s studies [1], only from the 1900s onwards has interest in cognitive functions become consistent in the scientific literature. A variety of reviews and editorials have yielded theoretical reflections on this topic, but less than 10% of articles (11% of screened articles in this review) report empirical evidence. The number of studies selected is small compared to those identified, highlighting the discrepancy between the speculative interest in this topic and the difficulty of applying a scientific method to assess the outcome of PP. In excluded studies, assessments of insight, metacognition, abstract reasoning, and mentalization have sometimes been used at baseline to clarify predictors of PP outcomes. Many of the identified studies involving patients with schizophrenia, other types of psychosis, or borderline disorders frequently assessed awareness, metacognition, mentalization, or insight, which may be affected by severe psychiatric disorders. In contrast, the low frequency of cognitive assessment in patients with neurotic disorders analyzed in this review may reflect the low interest of the patients themselves in this mental sphere, as, in fact, they undertake PP because of emotional distress, introspective maturation, psychosocial and adjustment problems, or other reasons, but not because they have a cognitive disorder. Therefore, this study broadens the scenario of cognitive functions in PP to non-psychotic and non-borderline disorders. The results also show that the designs, participants, and instruments vary among the selected studies, suggesting that inherent theoretical complexity and operational difficulty underlie this type of investigation. Moreover, while insight, emotion recognition, working memory, and executive functions were considered, other dimensions were largely neglected. Implicit cognitive functions were not assessed, although they had been highlighted by neurologists and psychoanalysts since the fin de siècle Victorian era, and later by Luria [63]. The link between the right hemisphere and unconscious processes and anosognosia or denial of illness has been historically reported, although not systematically, by authorities in neurology [64,65], and it was thought that the expression of hysterical symptoms in the left hemi-body implied the contribution of the right hemisphere in giving free rein to unconscious contents. Luria’s view on the role of the non-dominant hemisphere in the regulation of psychic processes emphasized the role of the frontal medio-basal regions and their connection with the superior brainstem and hypothalamus for the composition of the visceral brain, given their role in the regulation of cortical tone, impulses, and affective behavior [66]. Such classical observations and theoretical frameworks can shed light on the relationship between the right hemisphere and unconscious processing and the impact of PP on the functioning of this hemisphere and implicit functions.

### 4.2. Measurements Used to Evaluate the Cognitive Outcomes of Psychoanalytic Psychotherapy

Improvements were assessed by means of inventories, structured interviews, or rating scales compiled by the therapists or external examiners [35,36,40,41], but two studies [37,40] only used performance-based measures: tapping short-term memory and attention or interference control. This highlights the two main modes of assessment used in psychology and cognitive neurology, in particular the difference between self-assessed and neuropsychologically assessed cognitive functions. Self-assessed functions are highly dependent on the individual’s mood and self-esteem, which may prevent a completely objective assessment [67], and this may be particularly pronounced in patients with emotional problems. It can be argued that the combination of cognitive function-specific neuropsychological tests and self-assessment inventories can provide comprehensive information for baseline assessment and monitoring. It should be noted that cognitive functions are latent constructs, so they cannot be measured directly, and standardized instruments with metric and normative properties are required.

### 4.3. Functional Brain Changes After Psychoanalytic Psychotherapy

Twelve studies on controlled group designs or single cases, using fMRI, PET, SPECT, or EEG evaluations, showed changes in brain function after different types of PP. Neuroimaging documented that PP undertaken in single patients was associated with the activation of cortical and subcortical areas, particularly those included in or linked to the limbic system, and the alteration or normalization of neurotransmitters [47,48,49,50,51,52,53,54,55]. Changes in neural activity recorded by the EEG during PP have been useful for assessing different biological responses to therapy [56,57,58]. These results suggest that the study of brain connectivity holds promise for elucidating the architecture of the brain networks underlying PP. It has been debated whether neuroimaging methods are suitable for studying emotions, as the suboptimal environment of scanners may hinder the completeness of emotional processing. Temporal differences in the affective processes of the two hemispheres are lost with fMRI and PET, but can be captured with EEG and event-related potentials that offer greater temporal resolution. Thus, electrophysiological methods can capture emotional, physiological, behavioral, or autonomic aspects of psychic processes [68], as well as right hemisphere reactions to emotional events [69] and right amygdala responses to habituation [70], both of which are faster than their left counterparts. These aspects may have implications for monitoring PP-induced psychophysiological changes. However, the difficulty of recruiting long-term PP patients and involving them in invasive instrumental examinations may hinder scientific progress in this field.

Some shortcomings emerge from this State of the Art. Overall, the results may express an inherent tendency to reject measurements of cognition and brain outcomes for PP or a lack of interest in the neurobiological basis of PP, together with a poor connection between neurology and psychoanalysis. This gap in the scientific dialog seems to reflect the still-active philosophical impasse of Cartesian dualism, i.e., the view of psychological suffering as a state that has nothing to do with the neurophysiological basis of the human brain. A particularly open question is why to assess cognitive functioning in patients undergoing PP. It could be argued that widely used measures of cognition may not fully capture the kaleidoscopic complexity underlying individual and group PP processes, thus discouraging their clinical application. On the other hand, knowing patients’ mental resources before and during PP could provide therapists with an Olympian view of their condition, allowing them to identify the interplay between these resources and their role in shaping or interacting with defensive mechanisms, dreams, transference, and countertransference [4]. Cognitive resources, including motivational processes, are a prerequisite for successful therapy [35]. Their assessment can clarify the patient’s difficulties in participating in PP and their consequences on insights and the relationship between the patient and therapist [71]. In this sense, the cognitive neurology approach may also be necessary to decipher psychodynamic constructs and integrate them with neurophysiological and neuropsychological correlations.

### 4.4. Limitations of the Study

This review has inherent limitations, such as the impossibility of performing an in-depth analysis of the results, or a meta-analysis, which affects scoping reviews in general, and the results of this review in particular. Other limitations concern the way in which the articles were sought. It was not a simultaneous search in the same databases and lasted for a long time; some database tools were not used (e.g., MeSH in PubMed) and there was no researcher responsible for each database. These limitations, especially the long duration of the search, were due to the intention of not missing new articles and the difficulty in identifying studies despite the use of multiple keywords. It is also possible that the keywords were not sufficiently able to capture articles relevant to the review questions. However, the subject of this study is only one piece of a multifaceted topic in the unclear interface between PP and cognitive neurology, and its underlying thinking was to start with a focus on a circumscribed piece of knowledge.

## 5. Conclusions

This scoping review shows that the empirical approach has rarely been applied to assess the effects of PP on the brain and cognition. The results of the selected studies on neurotic and depressive disorders suggest that PP, particularly group PP, can stimulate certain cognitive functions and brain connectivity. This does not exclude the hypothesis that measurements of cognition and brain function may be, as partial aspects of a broader set of outcomes, indicators of the effectiveness or efficiency of PP.

## 6. Future Lines of Research

Further literature reviews using the same or different keywords or selection criteria, or comparing other clinical conditions, are needed to clarify the issues addressed in this study and provide an avenue for systematic reviews and empirical studies targeting PP. Collaboration between cognitive neurology and psychoanalysis seems indispensable to develop this field of research, which is only one piece of the broader research on PP outcomes and the interaction between PP and the brain.

## Figures and Tables

**Table 1:** Psychoanalytic individual psychotherapy.

Reference Country Study Design	Therapy Aim	Participants Diagnosis	Demographics	Cognitive Function Measurement	Other Measurements	Results
Kramer et al., 2018 [35] Switzerland. Naturalistic longitudinal	Manual-based short-term individual PP. Evaluate the relationship between the changes of biased thinking and symptomatic improvement	32 outpatients Adjustment disorders	Age: 20–39 years Women: 82% Education: not reported	Cognitive biases Cognitive Errors Rating Scale [38]. At session 4–7, At session 12–17 After session 20	SCL-90	Decrease of Abstraction score, increase of the Personalization scores and stability of total scores
Zeek et al., 2022 [36] Germany, Austria. Post-hoc data analysis of selected cases from a randomized trial	Individual PP Evaluate symptoms improvement and mentalization in patients with anorexia nervosa	28 outpatients Anorexia nervosa	Mean age: 28.7 years Women 100% Education not reported	Capacity to mentalize assessed by the In-Session-Reflective-Functioning-Scale [39]. At session 1–16 At session 17–32 At session 33–40	SIABS, SCID-I, PSR, EDI-2 PHQ-depression PHQ-9 HAQ (German version)	Mentalization capacity remained stable during individual PP and was associated with symptoms improvement in the last phase of the therapy

PP, psychoanalytic psychotherapy. SCL, Symptom Check List. SIABS, Structured Interview for Anorexic and Bulimic Syndromes. SCID-I, Interview for the assessment of comorbid conditions. PSR, Psychiatric Status Rating. EDI, Eating Disorder Inventory. PHQ, Patient Health Questionnaire. HAQ, Helping Alliance Questionnaire.

**Table 2:** Psychoanalytic group psychotherapy.

Reference Country Study Design	Therapy Aim	Participants Diagnosis	Demographics	Cognitive Function Measurements	Results
Forghani et al., 2016 [40] Iran Quasi-experimental study	Group PP with TA approach. Evaluate the effects of group PP on EI and executive functions in addicts	30 inpatients: 15 in group PP 15 in standard therapy. Drug-addicts	Not reported	Emotional intelligence Attention Emotional Quotient Inventory [42] Stroop test [43] At the beginning of PP After 12 sessions	Improvement of executive functions and emotional intelligence and decrease of drug dependency in patients treated with group PP

PP, psychoanalytic psychotherapy. TA, Transactional analysis.

**Table 3:** Individual psychoanalytic psychotherapy and group psychoanalytic psychotherapy.

Reference Country Study Design	Therapy Aim	Participants Diagnosis	Demographics	Cognitive Function Measurements	Other Measurements	Results
Klasik et al., 2012 [37] Poland Longitudinal and cross-sectional	Group PP combined with individual PP. Evaluate the effectiveness of different forms of therapeutic methods on the improvement of cognitive functions	60 inpatients: 20 treated with PP 20 treated with sertraline and PP 20 treated with sertraline Moderate depression	Not reported	Corsi and Signal Tests of the Attention Vienna Test System [44] At baselineAfter 8 weeks		Average improvement of short-term visual-spatial memory and selective attention after the therapies. Greater improvement after combined PP and sertraline
Werbart et al., 2016 [41] Sweden Longitudinal, prospective and naturalistic	Group PP or individual PP. Creating a typology of self-representations among young women and men in PP, to study longitudinal changes in self-representations, and to compare self-representations in the clinical sample with those of a non-clinical group	89 outpatients: 47 in individual PP 42 in group PP. Depression, anxiety, low-self-esteem, conflicts in close relationships or personality disorders. 23 healthy controls	Age: 18 to 25 years in the clinical and control group. Women: 36 in individual PP, 27 in group PP	Self-representations Blatt’s Object Relations Inventory [45] Differentiation-Relatedness Scale [46] At baselineAfter 1.5 years (termination) Three years after termination	SCL-90R Global Assessment of Functioning (DSM-IV)	Better balance between relatedness and self-definition and increased integration in self-representation in the clinical group. No tendency in the non-clinical group

PP, psychoanalytic psychotherapy. TA, Transactional analysis. SCL-90-R, Symptom Check list-90-Revised. DSM, Diagnostic Statistical Manual.

**Table 4:** Measurements of cognitive outcomes in studies on individual and group psychoanalytic psychotherapy.

Reference	Intrvention	Outcome	Tests	Test Material	Test Procedure	Evaluation Time
Kramer et al., 2018 [35] Individual PP	Short-term PP 12 therapists with at least 10-year experience Duration: up to a year Frequency: weekly sessions Total number of sessions: 24–48 with a mean of 34 sessions per patients	Insight	Cognitive errors rating scale [38]	Verbatim transcripts of interviews collected during therapy sessions	Transcripts’ rating by external examiners	Early: sessions 4–7: Mid-therapy: sessions 12–17 Close to the end: after the 20^th^ session
Zeek et al., 2022 [36] Individual PP	Focal PP and cognitive-behaviour therapy Duration: 10 months Frequency: 2 sessions a week Total number of sessions: 40 (28 patients included with a minimum of 26 sessions)	Reflective function	In-Session-Reflective-Functioning-Scale [39]	Verbatim transcripts of interviews collected during therapy sessions	Audiotapes of three phases of treatment (early, middle phase, and end) were transcribed anonymously based on the rules for transcription. The sessions were divided into sequences of 3 minutes, so a typical session (50 min) entailed about 17 text sequences	Early: sessions 1–16 Mid-therapy: sessions 17–32 End of therapy: sessions 33–40
Forghani et al., 2016 [40] Group PP	Group PP with TA approach Duration and frequency not specified Total number of sessions: 12	Emotional intelligence Executive functions Selective attention	Emotional Quotient [42] Stroop Test [43]	EQ Inventory: 133 items divided into 15 subscales grouped into: Intrapersonal EQ, Interpersonal EQ, Adaptability EQ, Stress Management EQ, General Mood EQ	EQ: Responses were based on a 5-point scale from “Definitely true for me” to “Not at all true for me.”. Stroop test: subjects were required to read words in three tables as fast as possible. In two tables with congruent stimuli, subjects had to read names of colors printed in black ink and to name different color patches. In one table with inconsistent ink colors, subjects had to name the color of the ink	Before therapy After 12 sessions
Klasik et al., 2012 [37] Group and Individual PP	Combined group and individual PP or pharmacotherapy Duration: 8 weeks Frequency: group PP: 1.5-hour session five times a week, individual PP: one-hour session twice a week. Total number of sessions: group PP: 40, individual PP: 16	Visual-spatial memory span Selective attention and visuospatial exploration	Corsi tapping test Signal Detection test (Vienna Test System) [44]	Wooden board with nine cubes Computer-based test system	Corsi tapping test: the examiner touches a cube at a time every two seconds, completing a sequence, the subject must touch exactly the same cubes in the same order of presentation immediately after the end of the sequence shown. Sequences of increasing length are presented. The score consists of the longest correctly played sequence or the correct nine-digit sequence. Vienna Test System: the subject is asked to identify the relevant signal in the presence of distractor signals on a pc screen.	At the beginning of the therapy After 8-weeks therapy
Werbart et al., 2016 [41] Group or Individual PP	Group or individual PP Mean duration (various): group PP: 16 months individual PP: 26 months (women for 22 months, men for 35 months) Clinical group: n = 41 Control group: n = 20 Mean duration (various): group PP: 16 months individual PP: 25 months Frequency of sessions (various): once or twice a week Total number of sessions: group PP: 2–85 individual PP: 1–51	Self-representation Differentiation between self and significant others	Object Relations Inventory [45] Differentiation-Relatedness Scale [46]	Interview 10-point scale	Subjects had to answer the question “Please provide a brief description of yourself”. The responses were rated on the D-R scale: -lack of basic differentiation between self and other (levels 1–2) -primitive forms of self and object representations (levels 3–5) -differentiated and integrated representations (levels 6–7) -mature and mutually related representation (levels 8–10).	At the beginning of the therapy At the end of the therapy 1.5 years later

PP, psychoanalytic psychotherapy. TA, Transactional Analysis.

## Data Availability

The original contributions presented in the study are included in the article, further inquiries can be directed to the corresponding author.
